# Important issues in developmental toxicity testing

**DOI:** 10.2478/v10102-010-0028-9

**Published:** 2010-11

**Authors:** Eduard Ujházy, Jana Navarová, Michal Dubovický, Štefan Bezek, Mojmír Mach

**Affiliations:** Institute of Experimental Pharmacology, Slovak Academy of Sciences, Dúbravská cesta 9, 841 04 Bratislava, Slovakia

Studies of individual development and its possible deterioration have been the concern since the 19th century, when Etienne Geoffroy de Saint-Hilaire (1772–1844) with his pioneer experiments opened the door for future experimental teratologists. Later scientists, focused on environmental agents which can alter embryonic and fetal development, such hyperthermia, malnutrition, pharmaceuticals, microbial toxins etc. Although the history of teratology involves many notable scientists, it has gained prominence after the big thalidomide tragedy in 1961. Principles of teratology were proposed later by James Wilson in his monograph *Environment and Birth Defects* (Wilson, 1973).

From the relative safety assessment point of view, it is essential to identify adverse agents, which can be harmful, related also to the development of the organism. The critical embryofetal developmental stages, when the agents are potentially most dangerous, require careful consideration as to maternal dosing regiments. Much of our present knowledge concerning the adverse effects of chemicals on mammalian development and particularly on human brain development relates to events during the early stages of gestation. Gestation is divided into two major periods, the embryonic and the fetal period. In humans the embryonic period constitutes 20% and the fetal 80% of the whole gestation period. In animals used frequently in research, such as mouse and rat, the opposite is seen: the embryonic period constitutes 80% and the fetal period 20% of the gestation.

In 1963, Werboff and Gottlieb came with the notion that chemical substances acting during sensitive prenatal brain development may cause behavioral alterations manifested in the postnatal period. They were the first to introduced the concept of behavioral teratology (Werboff and Gottlieb, 1963). Epidemiological as well as experimental studies in later periods showed that exposure of the developing organism to physical factors and/or chemical substances can cause not only structural but also functional anomalies. Functional maldevelopment of the brain can manifest as behavioral, emotional and/or cognitive disorders (Grandjean and Landrigan, 2006). Moreover, recent studies confirmed that any imbalance in the proper development of individual organs may result in their malfunctioning at various levels and in turn can result in chronic diseases in adulthood, such as diabetes, hypertension, etc.). (Barker, 2003). Not only chemical substances but also physical and environmental factors, such as insufficient nutrition, hypoxia, and stress, represent a serious risk factor for structural and functional development of the organism. In many cases, impairment of intrauterine development may cause the birth of a seemingly healthy individual with invisible stigma of future health problems.

## Asphyxia and Malnutrition as Developmental Disrupters

The important role of equilibrium of environmental factors during the embryofetal period is undisputable. Women of reproductive age are increasingly exposed to various environmental risk factors, such as prenatal viral infections, use of drugs, smoking, hypoxia, birth complications or stressful life events. Perinatal asphyxia is a major determinant of neurological morbidity and mortality in the neonatal period, causing long-term neurological complications, such as motor deficits including cerebral palsy, seizure and attention deficit/hyperactivity disorder (ADHD) (Hill and Volpe, 1989). The global use of medications that treat ADHD nearly tripled from 1993 to 2003 (Scheffler *et al*., 2007), suggesting wider understanding of the condition in the mental health community, healthcare coverage and the launch of ADHD-specific drugs. There is an emerging body of evidence correlating psychiatric disorders, schizophrenia in particular, to obstetric complications at the time of birth (Brixey *et al*., 1993; Günther-Genta *et al*., 1994). While a number of such complications have been identified as potential risk factors for schizophrenia, including breached birth, delayed labor, Caesarean (C)- section, or umbilical cord prolapse, the exact nature of the perinatal insult remains a matter of speculation. However, one consequence common to many of these perinatal complications is an episode of anoxia to the fetus.

Intrauterine growth restriction (IUGR) occurs when abnormal developmental processes prevent cells and tissues to grow and/or cause cells to decrease in size. This may happen when the fetus does not receive the necessary nutrients and oxygen needed for growth and development of organs and tissues, or because of infection or drug abuse. Although some babies are small due to genetics (their parents are small), in most cases IUGR is caused by other factors (maternal, embryofetal or placental). Recent epidemiologic studies report an inverse relationship between birth weight and hypertension, indicating that suboptimal fetal environment may also contribute to increased disease in later life (Ojeda *et al*., 2008). Findings of Costelo *et al*. (2007) suggest that adaptations in the womb optimizing survival under adverse conditions can lead to low birth weight and may later impair girls' ability to cope with stress. Their lower thresholds for stress-triggered illness may remain latent until they “encounter adversities that strain their capacity”.

## Experimental Approaches in Developmental Toxicology

In our experimental practice, we currently rely on animal tests to predict the potential for chemicals to cause reproductive harm in humans. It has been estimated that there are 65 000 chemicals in the general environment (Schardein, 1993) with hundreds or thousands of new chemicals being added to the environment each year. Only a few thousand of these chemicals have been examined for potential teratogenicity.

Animal tests for assessing reproductive toxicity are designed to cover the entire reproductive cycle, either as a series of tests that evaluate specific segments of the life cycle (reproduction/fertility, prenatal and postnatal development), or as a single protocol (two-generation test). However, testing for reproductive toxicity is animal-intensive. Several alternative methods have been proposed, driven by the efforts of 3Rs to replace, reduce and refine toxicity testing (Piersma, 1993), i.e. the use of established cell lines for developmental toxicity screening or rodent whole embryo culture. The usual protocol involves daily administration of the test substance to pregnant animals during their organogenic period and examination of the products of gestation shortly before parturition. Two species are required, in practice this is usually the rat and the rabbit, and three dosage levels, of which the highest dose causes some signs of maternal toxicity. However these tests (Segment I–III of reproductive toxicity testing) cover only the early period of life up to adulthood. Information on the delayed neurobehavioral consequences up to senescence is limited to a couple of reports. Therefore it is necessary to include in the animal testing also long-term studies or use alternative animal models, e.g. senescence-accelerated mouse strain (Yagi *et al*., 1988). Development of further up-to-date precautionary approaches that recognize the unique vulnerability of the developing organism are highly needed for testing and control of chemicals, drugs as well as other risk factors.
